# Monoclonal Antibody Therapies in Multiple Myeloma: A Challenge to Develop Novel Targets

**DOI:** 10.1155/2019/6084012

**Published:** 2019-11-03

**Authors:** Hiroko Nishida, Taketo Yamada

**Affiliations:** ^1^Department of Pathology, Keio University, School of Medicine, Tokyo 160-8582, Japan; ^2^Department of Pathology, Saitama Medical University, Faculty of Medicine, Saitama 350-0495, Japan

## Abstract

The treatment options in multiple myeloma (MM) has changed dramatically over the past decade with the development of novel agents such as proteasome inhibitors (PIs); bortezomib and immunomodulatory drugs (IMiDs); thalidomide, and lenalidomide which revealed high efficacy and improvement of overall survival (OS) in MM patients. However, despite these progresses, most patients relapse and become eventually refractory to these therapies. Thus, the development of novel, targeted immunotherapies has been pursued aggressively. Recently, next-generation PIs; carfilzomib and ixazomib, IMiD; pomalidomide, histone deacetylase inhibitor (HDADi); panobinostat and monoclonal antibodies (MoAbs); and elotuzumab and daratumumab have emerged, and especially, combination of mAbs plus novel agents has led to dramatic improvements in the outcome of MM patients. The field of immune therapies has been accelerating in the treatment of hematological malignancies and has also taken center stage in MM. This review focuses on an overview of current status of novel MoAb therapy including bispecific T-cell engager (BiTE) antibody (BsAb), antibody-drug conjugate (ADC), and chimeric antigen receptor (CAR) T cells, in relapsed or refractory MM (RRMM). Lastly, investigational novel MoAb-based therapy to overcome immunotherapy resistance in MM is shown.

## 1. Introduction

The treatment options in MM has changed dramatically over the past decade with the emergence of novel agents including proteasome inhibitors (PIs, bortezomib) and immunomodulatory drugs (IMiDs, thalidomide and lenalidomide) and exerts a remarkable impact on the outcome of MM patients [1–3]. However, most patients who achieve a prolonged response following initial therapy may ultimately relapse or become refractory. Thus, the development of novel, targeted immunotherapies has been pursued aggressively. Recently, next-generation PIs (carfilzomib and ixazomib) [4–9], IMiDs (pomalidomide) [10–12], histone deacetylase inhibitor (HDACi, panobinostat) [13–15], and the monoclonal antibodies (MoAbs, elotuzumab and daratumumab) have emerged and further improved the clinical outcome in MM patients who are refractory to prior treatments [12, 16–36]. Importantly, MM remains a chronic disease, so in order to overcome the disease relapse, ongoing challenges to pursue novel therapeutic strategies as well as predictive biomarkers for response or resistance to immunotherapies are required. Furthermore, these novel therapies are expected to be potentially useful in the treatment options for patients who are ineligible for autologous stem cell transplantation (SCT) followed by high-dose chemotherapy [37].

Monoclonal antibody (MoAb) therapies have been accelerating and shown to be able to improve the outcome of cancers [38]. In hematological malignancies, rituximab, a chimeric murine/human anti-CD20 monoclonal IgG_1*κ*_ antibody or of atumumab, a humanized anti-CD20 monoclonal IgG_1*κ*_ antibody, targeting CD20 on B cells, is currently indicated for the treatment of B-cell non-Hodgkin's lymphoma (NHL) and chronic lymphocytic leukemia (CLL). It exerts significant activity in combination with cytotoxic anticancer drugs [38, 39].

Although these progresses in immune therapies and their application for the treatment of MM have not succeeded until recently, these therapeutic strategies have finally attained a breakthrough with the development of the MoAb therapies targeting surface molecules, expressed in MM cells, such as elotuzumab, a humanized anti-CS1/SLAMF7 monoclonal antibody, and daratumumab, a humanized anti-CD38 monoclonal antibody, both of which have been approved in the treatment of relapsed or refractory MM (RRMM) patients who received at least three prior therapies including PIs and iMiDs [40–43]. Herein, we review an overview of the current status of MoAb therapies in RRMM. In addition, we introduce investigational novel MoAb therapies in RRMM and show future direction toward immunotherapy resistance in MM.

## 2. Monoclonal Antibodies (MoAbs) in MM

Potential MoAbs target various kinds of antigens including growth factors, signaling molecules, cell surface proteins, and molecule of adhesion. Ideally, these MoAb-therapeutic targets should be predominantly expressed on a majority of MM cells, but not on normal hematopoietic cells or nonhematopoietic tissues. MoAb therapies involve several mechanisms including direct cytotoxic effects, antibody-dependent cellular cytotoxicity (ADCC), complement-dependent cellular cytotoxicity (CDC), and interference with cell-to-cell interactions [40–43]. Other mechanisms include the use of intracellular toxins or radioactive isotopes conjugated to MoAbs after its internalization into tumor cells, which reveal cytotoxicity against tumor cells beyond those bearing MoAb target antigens [40–43].

### 2.1. CD20 and Rituximab

CD20 is a transmembrane phosphoprotein expressed on committed B lymphoid cells through the all stages of their development, but its expression is reduced in plasma cells. Rituximab, a chimeric murine/human anti-CD20 monoclonal IgG_1*κ*_ antibody targeting CD20 on B cells, is currently indicated for the treatment of B-cell non-Hodgkin's lymphoma (NHL) and chronic lymphocytic leukemia (CLL) [39]. It exerts significant activity in combination with cytotoxic anticancer drugs. However, CD20 is present only in a few plasma cells and is absent in most of plasma cells in MM. Therefore, few selected MM patients achieved only minimal responses (MD) [44–46]. Moreover, MM cells express increased levels of complement-inhibitory proteins which result in the reduction of CDC via rituximab against tumor cells.

### 2.2. CS1/SLAMF7 and Elotuzumab

Elotuzumab is a humanized IgG_1_ monoclonal antibody which targets SLAMF7, known as CS1, a glycoprotein, intensely expressed on MM cells and normal plasma cells as well as natural killer (NK) cells. It induces cytotoxicity against MM cells via NK cell-associated ADCC, NK cell activation, and inhibition of the interaction between MM cells and bone marrow stromal cells (BMSCs). Elotuzumab revealed intensive anti-MM efficacy and safety profiles when combined with IMiDs or PIs in previously treated RRMM [12, 16–21] (Table 1). The phase II results demonstrated that elotuzumab in combination with lenalidomide plus dexamethasone (Rd) in patients with RRMM showed safety and efficacy which was better than previously noted with Rd [17, 18]. Moreover, results of the phase III trial ELOQUENT-2 clearly proved the benefit of adding elotuzumab to Rd for the treatment of RRMM [18]. The overall response rates (ORRs) were 79% for the elotuzumab group and 66% for the control group; the PFS rate was 68 vs. 57% for the elotuzumab and control groups at 1 year and 41 vs. 27% at 2 years; the median PFS was 19.4 vs. 14.9 months for the elotuzumab and control groups [19]. Based on the results of these trials, elotuzumab attained food and drug administration (FDA) approval in 2015 in combination with Rd for the treatment of RRMM patients, who previously received two or three prior therapies. A phase III randomized study of Rd with or without elotuzumab in previously treated MM patients is currently ongoing. Phase II trials of elotuzumab plus pomalidomide and dexamethasone (EPd) vs Pd in 117 patients who received >2 prior therapies revealed that after a follow-up period of 9 months, EPd had a longer median PFS (10.3 vs 4.7 month) and a better ORR (53 vs 26%) [12]. Phase II trials of elotuzumab plus bortezomib and dexamethasone (EBd) vs Bd in 77 patients who had received one to three prior therapies showed that EBd had a longer median PFS (9.7 vs 6.9 months). However, there was no deference in ORR between EBd group and Bd group (66% vs 63%) [20, 21].

### 2.3. CD38 and Daratumumab

Daratumumab is a humanized IgG_1_-kappa monoclonal antibody targeting CD38, which is 46-kDa type II transmembrane glycoprotein, broadly expressed on plasma cells as well as lymphoid cells, myeloid cells, and nonhematopoietic tissues. It is also expressed in OCs. CD38 retains multiple functions including ectoenzymatic activity, signal transduction, and receptor-mediated regulation of cell adhesion [22, 23]. In preclinical studies, daratumumab revealed anti-MM cytotoxicity through multiple mechanisms including ADCC, ADCP, CDC, and direct apoptosis via FcR-mediated cross linking of daratumumab *in vitro* [24–26] (Table 2). Of note, no difference was revealed in daratumumab-associated ADCC or CDC between newly diagnosed and RRMM patients. The level of CD38 expression in MM cells was reported to be related to daratumumab-associated ADCC and CDC [24–26]. Moreover, daratumumab has several effects on the immune system. It increases CD8+/CD4+ and CD8+ Treg ratios as well as memory T cells, while decreasing naïve T cells, which enhance the overall immune response to MM cells [27].

Daratumumab revealed anti-MM efficacy as monotherapy as well as in combination with novel agents in heavily pretreated RRMM patients, which resulted in FDA approval in 2015. The GEN501 and SIRIUS trials demonstrated that daratumumab is active as monotherapy in RRMM patients [28, 29]. It showed improved ORRs regardless of refractoriness to prior therapies including PIs and IMiDs (31%). [30]. Phase III Castor trials revealed that daratumumab significantly improved ORR, PFS, and time to progression (TTP) in combination with Bd, ORR (83% vs 63%), the 12-month rate of PFS (61% vs 27%), and TTP at 12 months (65% vs 29%) [31]. Another phase III Castor study also revealed a significant benefit of D-Bd over Bd regardless of treatment history or cytogenetic risk [32]. Phase III POLLUX trials demonstrated remarkable efficacy of daratumumab in combination with lenalidomide plus dexamethasone (DRd) in patients with RRMM [33, 34]. The ORR was 92.9% in DRd group versus 72.9% in Rd group. DRd improved PFS compared with Rd with 12-month PFS rates of 83.2% in DRd group versus 60.1% in Rd group and 24-month PFS rate of 68.0% versus 40.9%, restrictively [33, 34]. The EQUULEUS study led to the FDA approval of daratumumab in combination with Pd in 2017 for RRMM patients who have received 2 or more prior line of therapy including lenalidomide and a PI. The median PFS was 8.8 months, the 12-month PFS rate was 42%, the median OS was 17.5 months, and the median 12-month survival rate was 66% [35].

## 3. Novel Target Antigens in MoAb Therapies in MM

### 3.1. CD38 and Isatuximab

Isatuximab is a chimeric IgG_1_-kappa anti-CD38 monoclonal antibody which selectively binds to a unique epitope on human CD38 receptor and elicits anti-MM activity by direct apoptosis, ADCC, and ADCP [47]. CDC was triggered in less than half of MM patients with high levels of CD38 in MM cells. A phase 1b open-label, dose escalation study showed that 57 patients who had received at least one prior line of therapy attained ORR of 52% by isatuximab plus Rd in 42 evaluable lenalidomide-refractory patients, and overall median PFS was 8.5 months [48]. Another phase 1b study of isatuximab plus Pd in patients with RRMM who had received more than 2 prior therapies also revealed that ORR was 62%; median duration of response was 18.7 months; and PFS was 17.6 months [49].

### 3.2. Interleukin-6 (IL6) and Siltuximab

Interleukin-6 is an important cytokine for the growth and survival of MM cells. It is chiefly produced by BMSCs and increased by several cytokines. A chimeric anti-IL-6 antibody, siltuximab, revealed cytotoxicity in MM patients who was refractory to dexamethasone [50]. In addition, it increased cytotoxicity with Bd in combination, whereas in a phase 2 randomized study of siltuximab plus bortezomib, the addition of siltuximab to bortezomib did not appear to improve PFS or OS in refractory MM patients [51]. The other study showed that there were no responses to siltuximab but combination therapy with dexamethasone yielded a partial or minimal response rate of 23%, in dexamethasone-refractory MM [51].

### 3.3. PD-1/PD-L1 Inhibitors

Programmed cell death protein 1 (PD-1)/programmed cell death ligand 1 (PD-L1) pathway is a negative regulator of immune activation [52]. Recently, there are discrepancies concerning programmed death PD-L1 expression on plasma cells in MM. Several data demonstrated that PD-L1 is overexpressed on MM plasma cells but not on normal plasma cells [53–56]. It was reported that PD-L1 expression on plasma cells was associated with increased risk of progression from smoldering MM (SMM) into MM [57], whereas other reports showed that no difference was detected in PD-L1 expression on plasma cells between MM, SMM, monoclonal gammopathy of undetermined significance (MGUS), and healthy individuals [58, 59]. Similarly, discordant results were reported regarding PD-1 expression on immune cells, including T cells and NK cells in MM. Paiva et al. showed that PD-1 was overexpressed on CD4+ and CD8+ T cells in MM patients [58]. Benson et al. demonstrated that PD-1 expression was increased on NK cells from MM patients, compared with normal NK cells, whereas Paiva et al. demonstrated there was no difference between these cells [58, 60].

Among hematological malignancies, antibody blockade of the PD-1/PD-L1 pathway is a highly effective therapeutic approach for patients with classical Hodgkin lymphoma, 97% of which typically exhibits an overexpression of PD-L1 due to the alteration in chromosome 9p24.1 (54). Therefore, the PD-1/PD-L1 axis is a good target for MoAbs, leading immune cells to kill tumor cells. The use of nivolumab, a human IgG4 MoAb which blocks the interaction with PD-L1 and PD-L2 by binding to the PD-1 receptor on activated immune cells, was approved by FDA in 2016 for the treatment of relapsed or progressed Hodgkin lymphoma [52]. However, the outcome of checkpoint blockade by monotherapy with PD-1/PD-L1 inhibitors is unsatisfactory in MM, compared with solid tumors due to the reduced immune dysfunction in MM [58, 59]. In contrast, lenalidomide enhances the effect of PD-1/PD-L1 blockade on both T cell- and NK cell-mediated cytotoxicity. The combination therapy of lenalidomide plus PD-1/PD-L1 inhibitors increased interferon *γ* by BM-derived effector cells in MM and was associated with increased apoptosis of MM cells, suggesting synergistic cytotoxic effects [56, 61, 62]. There are only limited data from clinical trials of PD1/PDL1 MoAbs in MM patients. The phase Ib trial of nivolumab monotherapy in 27 RRMM patients showed the stabilization of disease status in 17 patients, lasting a median of 11.4 weeks [63]. A phase I study of pembrolizumab with Rd in RRMM patients revealed a partial response rate of 50% [61, 62, 64, 65]. A phase 3 study of the combination of Rd with or without pembrolizumab was performed in transplant ineligible newly diagnosed MM patients (KEYNOTE-185 trial) [61, 62, 64]. A Phase 3 study of the combination of Pd with or without pembrolizumab was conducted in the KEYNOTE-183 trial, and it led FDA to discontinue the trial, due to increased risk of death of patients [61, 62, 65].

### 3.4. Bispecific T-Cell Engager (BiTE) Antibodies (BsAb)

Bispecific T-cell engager (BiTE) antibodies (BsAbs) are constructs, composed of 2 linked MoAbs which target 2 epitopes. One arm of antibody, scFvs, binds to CD3 on tumor-specific T cells, while the other arm binds to tumor-specific antigen on tumor cells [66, 67]. Cross linkage of T cells to the tumor cells causes T cells to release cytotoxic molecules such as perforin, which creates transmembrane pores in tumor cells, and granzyme B, which initiates apoptosis toward tumor cells. In addition, cytokine production from T cells activates its proliferation to kill tumor cells. BsAbs are characterized by small size (5 kDa), which induces high efficacy toward tumor cells, but its serum half-life is short [66, 67]. B-cell maturation antigen (BCMA) belongs to tumor necrosis factor superfamily member 17, also named “TNFRSF17 or CD269,” which is uniformly expressed in malignant plasma cells but not in normal essential nonhematopoietic tissues, and only restricted expression is detected in normal hematopoietic cells including normal plasma cells and mature B lymphocytes. Thus, it is a highly plasma cell specific antigen and has a central role in regulating B-cell maturation and differentiation into plasma cells by engaging a proliferation-inducing ligand (APRIL) cells. This expression pattern leads to the development of BCMA-specific mAbs, BsAbs, antibody-drug conjugates (ADCs), and chimeric T cell receptor (CAR) T cells [68–70]. BsAb, BI-836909 (AMG420), the first bispecific scFv, simultaneously binds to CD3+ T cells and BCMA + MM cells which make a cross linking between both cells to induce cytolytic synapse, activate T cells, and lyse BCMA + MM cells. In phase I study in RRMM patients, it exhibited potent and high efficacy by depleting BCMA + MM cells [68–70]. CD3xCD38 BsAb, engineered to direct T cells to CD38 on tumor cells, was also developed. The phase 1 multicenter study of GBR1342 is underway [71].

### 3.5. Antibody-Drug Conjugates (ADCs)

Antibody-drug conjugate is composed of recombinant MoAbs, bound to cytotoxic chemical agents through synthetic chemical linkers. MoAbs bind to the cell surface antigen on tumor cells and are internalized with the chemicals. Thus, the cytotoxic chemicals are released and transported from lysosome into cytosol to kill tumor cells [72]. GSK2857916 is a humanized and IgG_1_ MoAb with high affinity to BCMA with afucosylated Fc linked to auristatin F noncleavable linker, maleimidocaproyl. In preclinical study, it binds to BCMA + MM cells and induces G2/M arrest and apoptosis by the activation of caspase 3/7 and 8. The naked form of ADC augmented effector-mediated cytotoxicity including ADCC and ADCP against patient MM cells [72]. In MM xenograft models, GSK2857916 depletes MM cells but surrounding BCMA-BM accessory cells remain unharmed. Its cytotoxicity is further increased by GSK2857916 plus lenalidomide in combination. In phase 1 study of GSK2857916 in RRMM patients, GAK2857916 monotherapy revealed a 60% response rate and median PFS of 7.9 months [73, 74]. Anti-BCMA approaches, alone or in combination with iMIDs or immune checkpoint inhibitors, will be evaluated in clinical trials in MM [70].

### 3.6. Chimeric Antigen Receptor (CAR) T Cells

CARs are fusion proteins incorporating an antigen-recognition domain and T-cell signaling domain. T cells are genetically modified to express CARs, which specifically recognize target antigens on tumor cells [75–77]. CAR T-cell therapy has already approved by FDA and European Medicine Agency (EMA) for the treatment of relapsed of refractory B- acute lymphoblastic leukemia (ALL) and diffuse large B cell lymphoma (DLBCL) [75–77]. CAR-expressing T cells targeting CD19 revealed efficacy in patients with acute lymphoblastic leukemia (ALL) or B-cell NHL. This success of CAR-T cells against leukemia or lymphoma has encouraged the development of CAR-T therapies for MM. In the first human clinical trials, Carpenter et al. designed the first novel CAR targeting BCMA in MM and demonstrated CAR-BCMA T cells had powerful activity against MM that was resistant to standard therapies [78, 79]. Moreover, bb2121 was produced by transducing autologous T cells with a lentiviral vector encoding a second-generation CAR incorporating an anti-BCMA single-chain variable fragment, CD137 costimulatory motif, and a CD3-zeta signaling domain [80]. A phase 1 clinical study of bb2121 in heavily pretreated RRMM patients revealed that 85% of the patients had a clinical response lasting a median of 10.9 months without any ongoing MM therapies [80]. Currently, CAR-T cell therapy for MM remains experimental. CAR-T cell therapy is a potentially life-threatening therapeutic approach, which needs to be administrated in experience hospitals. Now, phase 3 trials are just starting for RRMM in 2019. In addition, novel CARs targeting alternative plasma cell antigens including CD38, CD44v6, and SLAMF7(CS) are being developed [81, 82].

## 4. Experimental Research in Novel MoAb Therapy in RRMM

### 4.1. Investigational MoAbs

Target antigens for MoAb are either cell surface membrane proteins or soluble factors including cytokines or chemokines expressed or secreted in MM cells. Their functions include MM cell growth, cellular adhesion, angiogenesis, apoptosis, and cell-to-cell contact between MM cells microenvironmental cells. Investigational mAbs targeting CD138, CD56, CD40, CD74, BAFF, BCMA, GRP78, IGF-1R, and ICAM-1 are preclinically developed, and several of them are in clinical trials [83–92] (Table 3).

### 4.2. Humanized Anti-CD26 Monoclonal Antibody (huCD26mAb)

**Figure 1:**
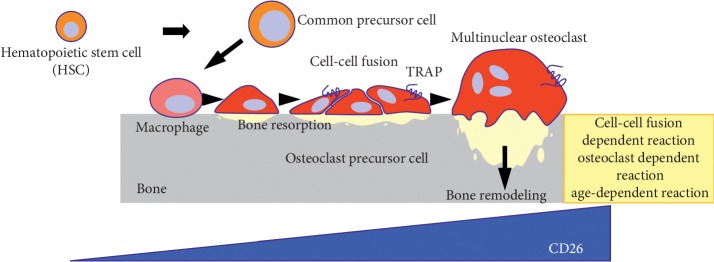
CD26 in human osteoclast development CD26 expression is increased during human osteoclast (OC) development.

**Figure 2:**
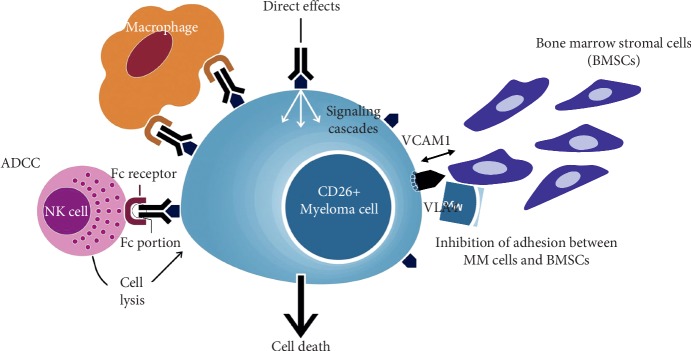
Humanized anti-CD26 monoclonal antibody (huCD26mAb): mechanisms of action huCD26mAb inhibits CD26 + MM cell growth chiefly via ADCC.

CD26 is a 110 kDa transmembrane glycoprotein with dipeptidyl peptidase (DPPIV) activity, which is widely expressed in various normal cells such as T lymphocytes, natural killer (NK) cells, basophils, eosinophils, endothelial cells, and epithelial cells [93–96]. In addition, CD26 is expressed in several tumor cells including malignant lymphoma, mesothelioma, renal cell carcinoma, and hepatocellular carcinoma and is involved in T-cell activation and tumorigenesis [97, 98]. We have recently characterized CD26 as a potential therapeutic target for the treatment of MM [99]. We identified CD26 expression in human osteoclasts (OCs) in healthy individuals (Figure 1). Its expression is further increased in osteoclasts in osteolytic bone tumors including MM, adenocarcinoma, lung cancer, and osteosarcoma. huCD26mAb, a humanized IgG_1_ monoclonal antibody that directly targets CD26, inhibits human OC differentiation *in vitro* and *in vivo* analysis [99]. In the bone marrow tissue of MM patients, we found that CD26 was present in plasma cells around OCs or endothelial cells. *In vitro* immunostaining or flow cytometry studies revealed that although CD26 expression was low or absent on MM cell lines cultured alone, it was intensely and uniformly expressed on MM cell lines cocultured with OCs [100]. The augmented CD26 expression in MM cells was exploited to enhance cytotoxicity of huCD26mAb chiefly via a substantial increase in antibody-dependent cytotoxicity (ADCC) against MM cells, direct effects or inhibition of the adhesion between MM cells and BM stromal cells (BMSCs) (Figure 2). Moreover, huCD26mAb in combination with the existing standards of care including bortezomib and lenalidomide synergistically enhanced huCD26mAb-induced ADCC activity against CD26 + MM cells compared with each agent alone [100]. Lastly, therapeutic effect of huCD26mAb against MM cell growth and its related osteolytic lesion was also validated *in vivo*, using a xenograft model: an intrabone tumor model of MM. Our preclinical results demonstrated that huCD26mAb elicited significant anti-MM efficacy by impairing both CD26 + MM cells and OCs *in vivo*, suggesting that CD26 could be an ideal therapeutic target of antibody-based therapy in RRMM [100].

## 5. Conclusion

During the last decades, therapeutic strategies in MM have dramatically changed. MoAbs act synergistically with backbone regimens including iMIDs, PIs, or HDACi and have benefits to overcome resistance to prior therapies. The future treatment options of MM to overcome resistance are promising by combination with MoAbs plus these novel agents, check point inhibitors or CAR T-cell therapy.

## Figures and Tables

**Table 1:** Summary of clinical trials in anti-CS1/SLAMF7 antibody in relapsed/refractory MM.

References		Phase	Regimen	ORR (%)	PFS (mo)	OS
Richardson et al. [17]		2	Elo + Rd	84.00%	NA	NA
Lonial et al. [18]	ELOAUENT2	3	Rd ± Elo	79% vs 66%	19.4 mo vs 14.9 mo	NA
Dimopoulos et al. [12]		2	Pd ± Elo	53% vs 26%	10.3 mo vs 4.7 mo	NA
Jakubowiak et al. [20]	Elo-Bd	2	Bd ± Elo	66% vs 63%	9.7 mo vs 6.9 mo	1 yr 85% vs 74%
Zonder et al. [16]	Phase1 Elo	1	Elo Dose Escalation	MTD not identified	NA	NA
Jakubowiak, et al. [21]	Elo-Bd	1	Elo + Bd	48.00%	9.5 mo	NA
Lonial, et al. [19]	Elo-Rd	1	Elo + Rd	82.00%	NA	NA

MM, multiple myeloma; Elo, elotuzumab; Rd, lenalidomide plus dexamethasone; Pd, pomalidomide plus dexamethasone; Bd, bortezomib plus dexamethasone, NA, not available; MTD, maximum tolerated dose.

**Table 2:** Summary of clinical trials in anti-CD38 antibody in relapse/refractory MM.

References		Phase	Regimen	ORR (%)	PFS (mo)	OS
Lokhorst et al. [28]	GEN501	1/2	Dara monotherapy	36%	5.6 mo	1 yr 77%
Lonial et al. [29]	SIRIUS	2	Dara monotherapy	17%	3.7 mo	1 yr 65%
Spencer et al. [32]	CASTOR	3	Bd ± Dara	83% vs 63%	1.5 yr 48% vs 8%	NA
Palumbo et al. [31]	CASTOR	3	Bd ± Dara	83% vs 63%	1 yr 61% vs 27%	NA
Dimopoulos et al. [33]	POLLUX	3	Rd ± Dara	93% vs 76%	1 yr 83% vs 60%	NA
Dimopoulos et al. [34]	POLLUX		Rd ± Dara	93% vs76%	2 yr 68% vs 41%	NA
Chari et al. [35]	EQULLEUS	1b	Pd ± Dara	60%	1 yr 42%	1 yr 89%

MM, multiple myeloma; Dara; daratumumab, Rd, lenalidomide plus dexamethasone; Bd, bortezomib plus dexamethasone; Pd, pomalidomide plus dexamethasone; NA, not available; MTD, maximum tolerated dose.

**Table 3:** Investigational monoclonal antibodies in MM.

Target molecule	mAb	Type	Clinical trials
CD138	Indatuximab ravtansine	ADC	Inda ± Rena ORR 78% vs 4%
CD56	Lorvotuzumab	ADC	Lorv+/Rd ORR 56% vs 7%
CD40	Dacetuzumab, lucatumumab	Humanized	Luc; 4% attained prolonged PR
CD74	Milatuzumab	Humanized	No objective responses
BAFF	Tabalumab	Humanized	Bd + Taba; ORR 44%
BCMA	GSK2857916	ADC	MTD not determined
GRP78	PAT-SM6	Humanized	MTD not determined
IGF-1R	AVE1642	Humanized	No objective responses
ICAM-1	BI-505	Humanized	No objective responses
CD26	YS110 (huCD26mAb)	Humanized	Best responses 50%

ADC, antibody-drug conjugate; Lena, lenalidomide; Inda, indatuximab ravatansine, Rd, lenalidomide plus dexamethasone; Lorv, lorvotuzumab; Luc, lucatumumab; PR, partial response; Bd, bortezomib + dexamethasone; Taba, tabalumab; MTD, maximum tolerated doses.

## Supplementary Materials

Supplementary Materials CD26 in human osteoclast development humanized anti-CD26 monoclonal antibody (huCD26mAb): mechanisms of action summary of clinical trials in anti-CS1/SLAMF7 antibody in relapsed/refractory MM. Summary of clinical trials in anti-CD38 antibody in relapsed/refractory MM. Investigational monoclonal antibodies in MM.
