# Supplementary material for: Monoclonal Antibody Therapies in Multiple Myeloma: A Challenge to Develop Novel Targets
Source: J Oncol. 2019 Nov 3;2019:6084012. doi: 10.1155/2019/6084012 (PMC6875016; doi:10.1155/2019/6084012)
Supplement: Supplementary Materials — CD26 in human osteoclast development humanized anti-CD26 monoclonal antibody (huCD26mAb): mechanisms of action summary of clinical trials in anti-CS1/SLAMF7 antibody in relapsed/refractory MM. Summary of clinical trials in anti-CD38 antibody in relapsed/refractory MM. Investigational monoclonal antibodies in MM. [file 6084012.f1.pptx]

## Slide 1
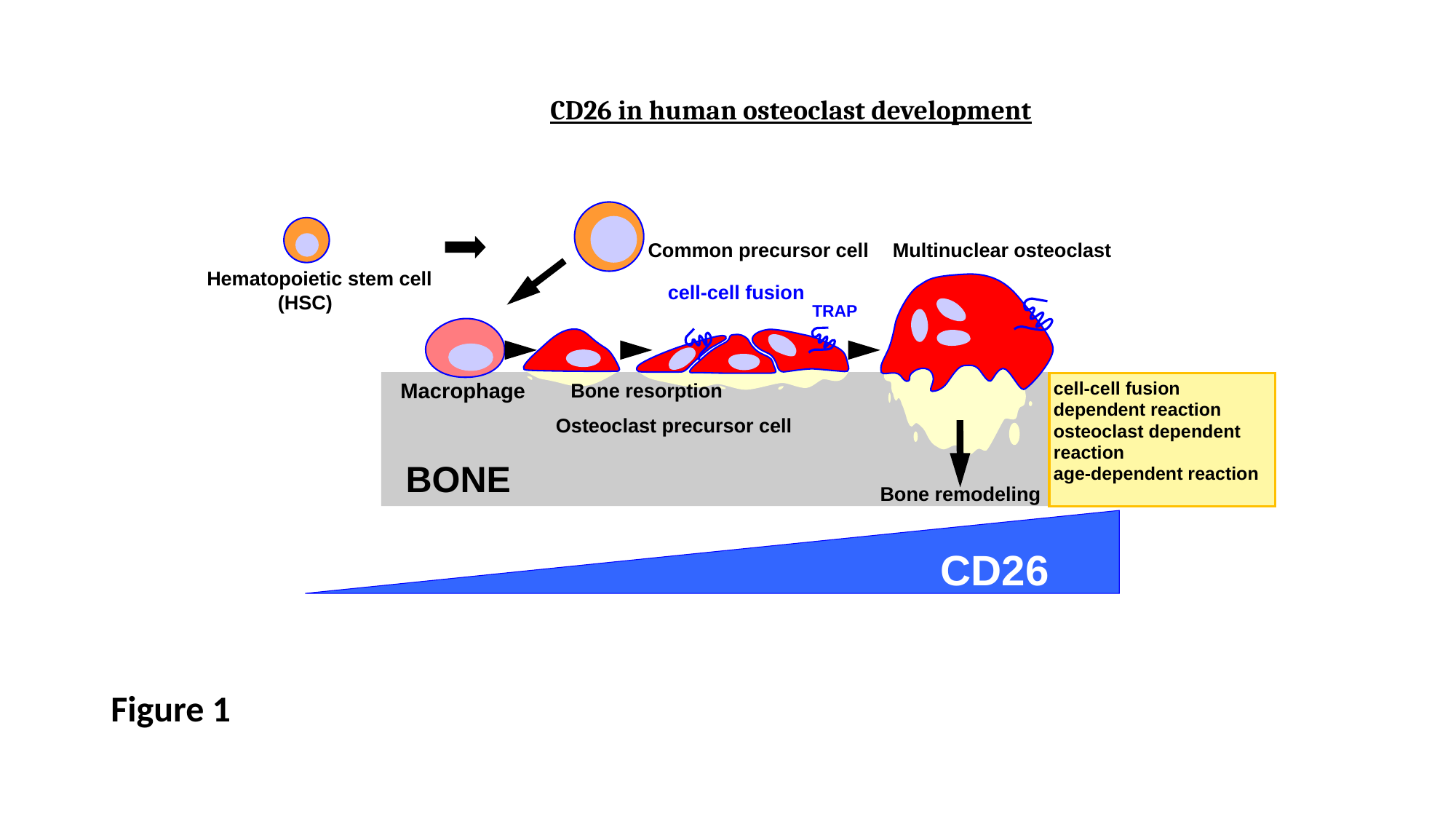

CD26 in human osteoclast development
CD26
Multinuclear osteoclast
Common precursor cell
Hematopoietic stem cell
 (HSC)
cell-cell fusion
TRAP
Macrophage
cell-cell fusion dependent reaction osteoclast dependent reaction
age-dependent reaction
macrophage
Bone resorption
Osteoclast precursor cell
BONE
Bone remodeling
CD26
Figure 1

## Slide 2
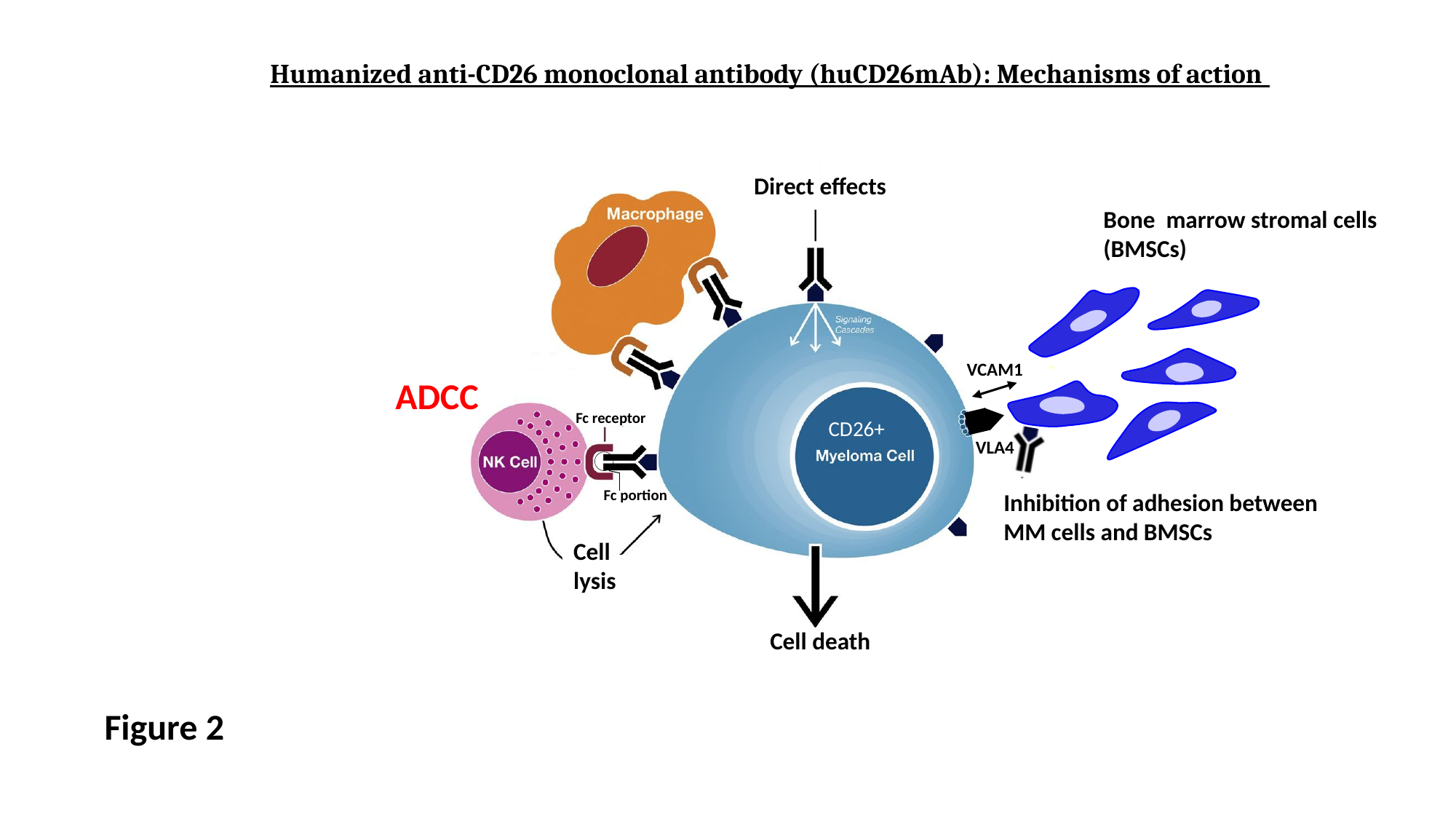

Humanized anti-CD26 monoclonal antibody (huCD26mAb): Mechanisms of action
Direct effects
Bone marrow stromal cells
(BMSCs)
VCAM1
ADCC
Fc receptor
CD26+
VLA4
Fc portion
Inhibition of adhesion between
MM cells and BMSCs
Cell
lysis
Cell death
Figure 2

## Slide 3
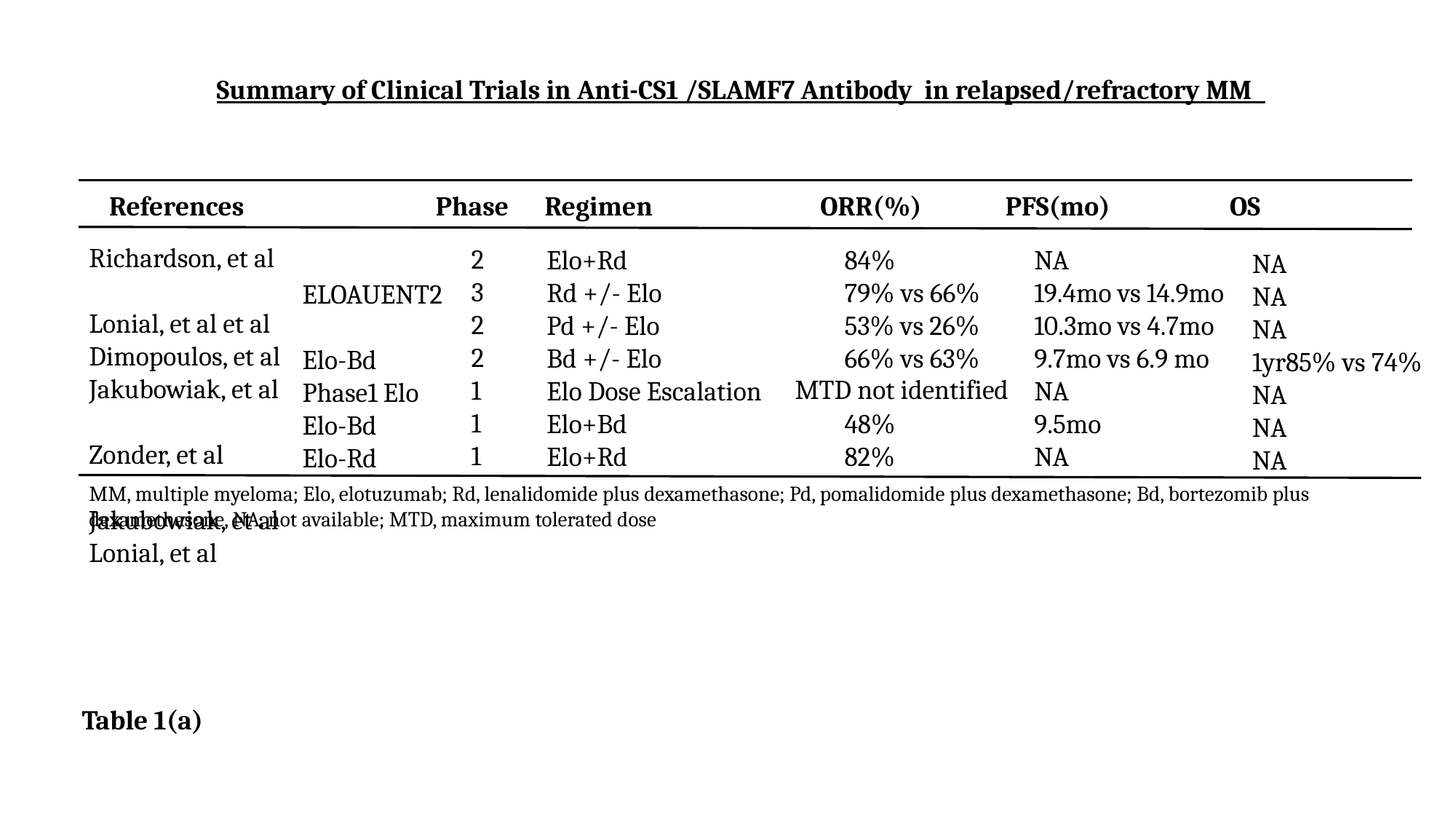

Summary of Clinical Trials in Anti-CS1 /SLAMF7 Antibody in relapsed/refractory MM
References Phase Regimen ORR(%) PFS(mo) OS
Richardson, et al
Lonial, et al et al
Dimopoulos, et al
Jakubowiak, et al
Zonder, et al
Jakubowiak, et al
Lonial, et al
2
3
2
2
1
1
1
Elo+Rd
Rd +/- Elo
Pd +/- Elo
Bd +/- Elo
Elo Dose Escalation
Elo+Bd
Elo+Rd
84%
79% vs 66%
53% vs 26%
66% vs 63%
48%
82%
NA
19.4mo vs 14.9mo
10.3mo vs 4.7mo
9.7mo vs 6.9 mo
NA
9.5mo
NA
NA
NA
NA
1yr85% vs 74%
NA
NA
NA
ELOAUENT2
Elo-Bd
Phase1 Elo
Elo-Bd
Elo-Rd
MTD not identified
MM, multiple myeloma; Elo, elotuzumab; Rd, lenalidomide plus dexamethasone; Pd, pomalidomide plus dexamethasone; Bd, bortezomib plus dexamethasone, NA, not available; MTD, maximum tolerated dose
Table 1(a)

## Slide 4
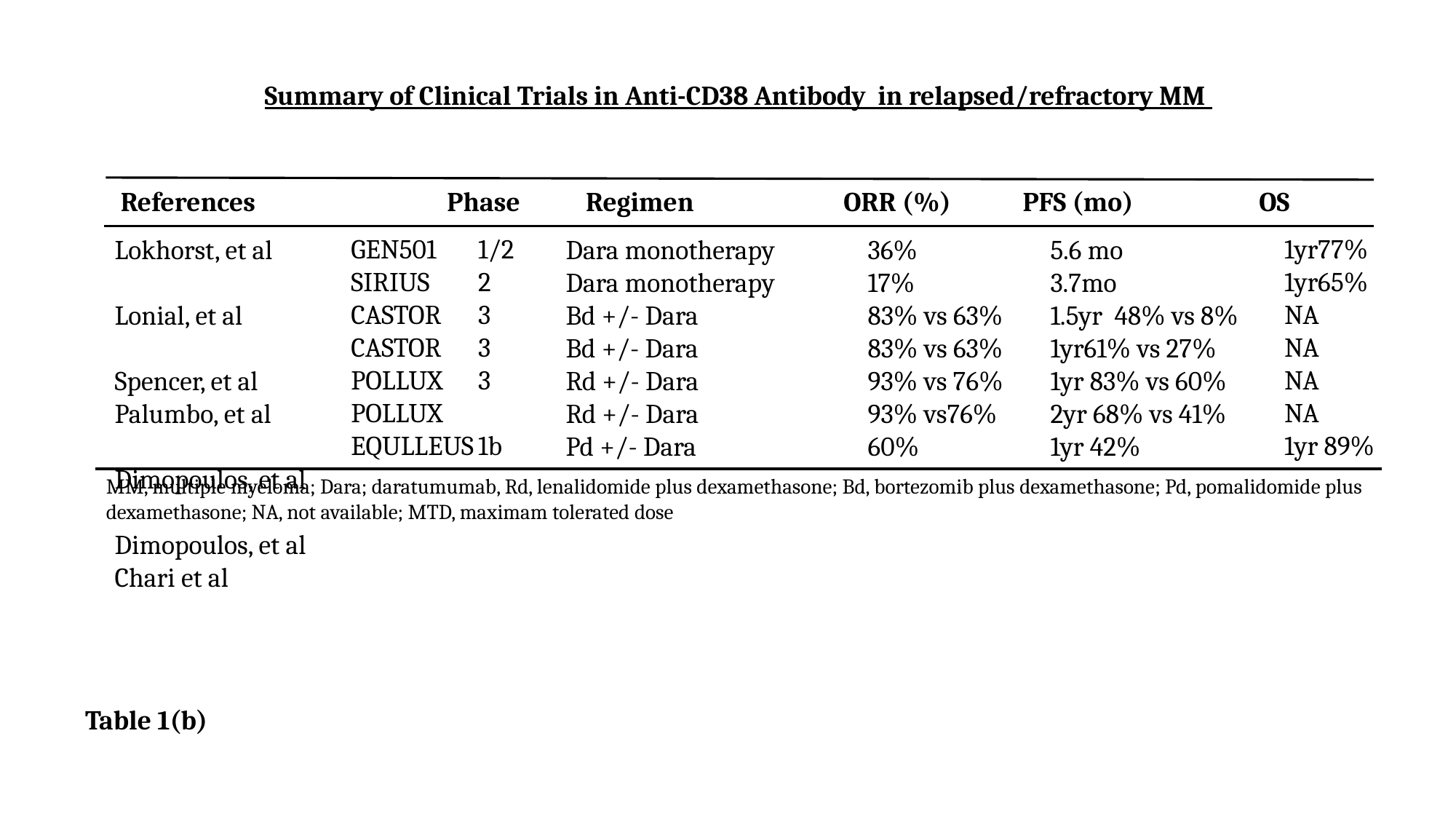

Summary of Clinical Trials in Anti-CD38 Antibody in relapsed/refractory MM
 References Phase Regimen ORR (%) PFS (mo) OS
GEN501
SIRIUS
CASTOR
CASTOR
POLLUX
POLLUX
EQULLEUS
1/2
2
3
3
3
1b
1yr77%
1yr65%
NA
NA
NA
NA
1yr 89%
Dara monotherapy
Dara monotherapy
Bd +/- Dara
Bd +/- Dara
Rd +/- Dara
Rd +/- Dara
Pd +/- Dara
36%
17%
83% vs 63%
83% vs 63%
93% vs 76%
93% vs76%
60%
5.6 mo
3.7mo
1.5yr 48% vs 8%
1yr61% vs 27%
1yr 83% vs 60%
2yr 68% vs 41%
1yr 42%
Lokhorst, et al
Lonial, et al
Spencer, et al
Palumbo, et al
Dimopoulos, et al
Dimopoulos, et al
Chari et al
MM, multiple myeloma; Dara; daratumumab, Rd, lenalidomide plus dexamethasone; Bd, bortezomib plus dexamethasone; Pd, pomalidomide plus dexamethasone; NA, not available; MTD, maximam tolerated dose
Table 1(b)

## Slide 5
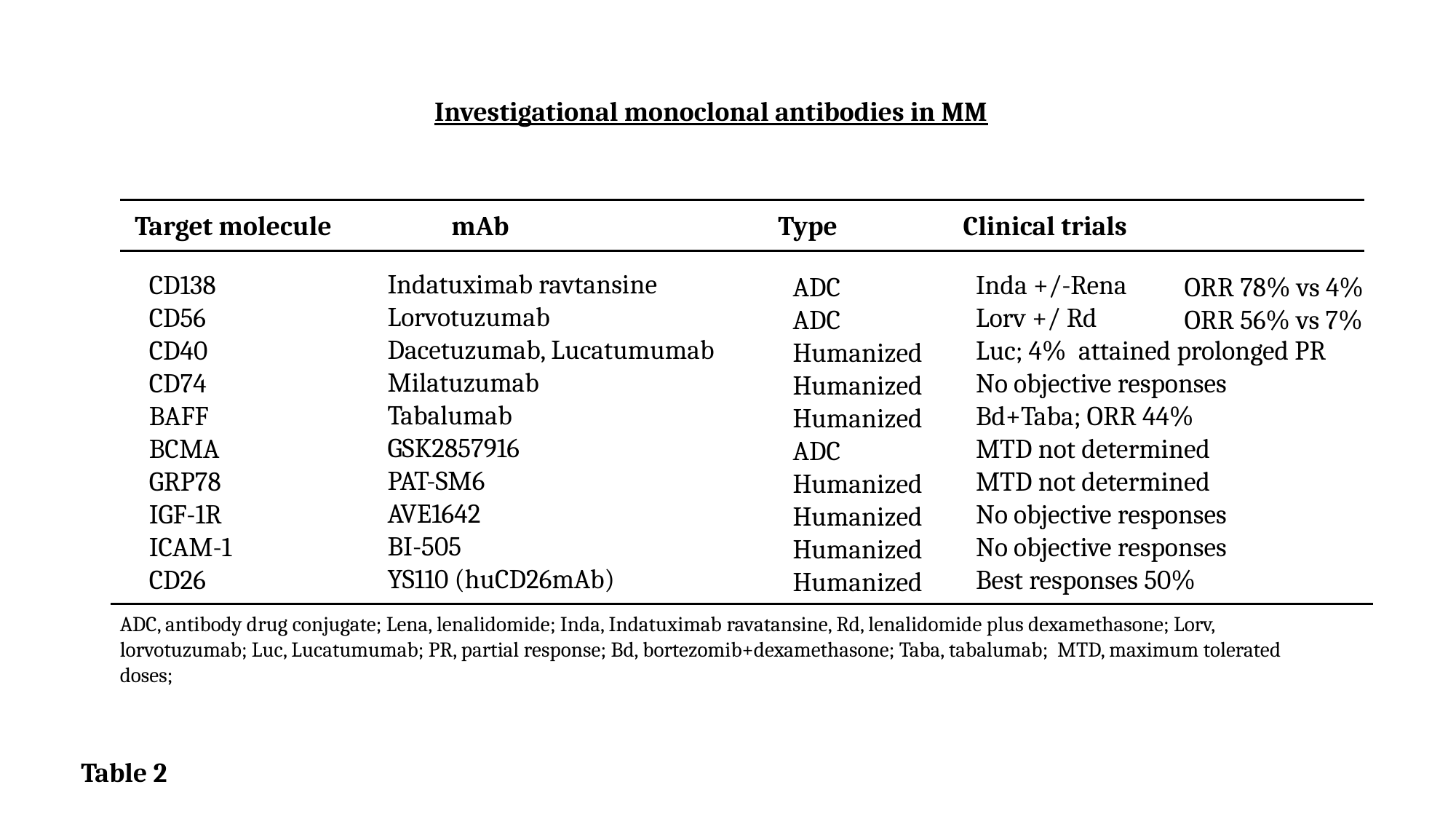

Investigational monoclonal antibodies in MM
Target molecule mAb Type Clinical trials
Indatuximab ravtansine
Lorvotuzumab
Dacetuzumab, Lucatumumab
Milatuzumab
Tabalumab
GSK2857916
PAT-SM6
AVE1642
BI-505
YS110 (huCD26mAb)
CD138
CD56
CD40
CD74
BAFF
BCMA
GRP78
IGF-1R
ICAM-1
CD26
Inda +/-Rena
Lorv +/ Rd
Luc; 4% attained prolonged PR
No objective responses
Bd+Taba; ORR 44%
MTD not determined
MTD not determined
No objective responses
No objective responses
Best responses 50%
ADC
ADC
Humanized
Humanized
Humanized
ADC
Humanized
Humanized
Humanized
Humanized
ORR 78% vs 4%
ORR 56% vs 7%
ADC, antibody drug conjugate; Lena, lenalidomide; Inda, Indatuximab ravatansine, Rd, lenalidomide plus dexamethasone; Lorv,
lorvotuzumab; Luc, Lucatumumab; PR, partial response; Bd, bortezomib+dexamethasone; Taba, tabalumab; MTD, maximum tolerated doses;
Table 2
